# *“Gnothi Seauton”*: Leveraging the Host Response to Improve Influenza Virus Vaccine Efficacy

**DOI:** 10.3390/vaccines6020023

**Published:** 2018-04-12

**Authors:** Hannah D. Stacey, Neda Barjesteh, Jonathan P. Mapletoft, Matthew S. Miller

**Affiliations:** Michael G. DeGroote Institute for Infectious Diseases Research, McMaster Immunology Research Centre, Department of Biochemistry and Biomedical Sciences, McMaster University, Hamilton, ON L8S 4K1, Canada; staceyhd@mcmaster.ca (M.D.S.); barjestn@mcmaster.ca (N.B.); Mapletoj@mcmaster.ca (J.P.M.)

**Keywords:** influenza virus, vaccines, host factors, systems biology, polymorphisms, dendritic cells, adjuvants, innate immunity, adaptive immunity

## Abstract

Vaccination against the seasonal influenza virus is the best way to prevent infection. Nevertheless, vaccine efficacy remains far from optimal especially in high-risk populations such as the elderly. Recent technological advancements have facilitated rapid and precise identification of the B and T cell epitopes that are targets for protective responses. While these discoveries have undoubtedly brought the field closer to “universal” influenza virus vaccines, choosing the correct antigen is only one piece of the equation. Achieving efficacy and durability requires a detailed understanding of the diverse host factors and pathways that are required for attaining optimal responses. Sequencing technologies, systems biology, and immunological studies have recently advanced our understanding of the diverse aspects of the host response required for vaccine efficacy. In this paper, we review the critical role of the host response in determining efficacious responses and discuss the gaps in knowledge that will need to be addressed if the field is to be successful in developing new and more effective influenza virus vaccines.

## 1. Introduction

Since Edward Jenner’s famous publication of *An Inquiry into the Causes and Effects of the Variolae Vaccinae* in 1798, which described the heterologous immunity mediated by the cowpox virus infection against the smallpox virus, the field of vaccinology has had a decidedly antigen-centric focus. Most early vaccines were produced by either killing/inactivating or attenuating a pathogen in an effort to trigger immunity in the absence of severe infections [1]. This was done with little regard for how host factors/pathways might influence outcome. In the context of the influenza virus, both inactivated and live-attenuated vaccine formulations have been approved for some time. Tremendous effort has been exerted to identify the specific B cell and/or T cell epitopes that contribute the most to protect immune responses. This knowledge has catalyzed an explosion in vaccine platforms including subunit vaccines (e.g., Flublok^®^), DNA vaccines, and live vector vaccines. However, our understanding of the host factors and pathways (beyond B cell and T cell responses) that are important for efficacious vaccine responses is only beginning to take shape [2].

The need to better understand the host response is clearly illustrated by the observation that a given vaccine formulation often elicits a wide spectrum of responses, as measured by antibody titers or T cell frequencies, across the population. Factors including age, sex, and underlying medical conditions (e.g., obesity) are all known to affect vaccine efficacy [3,4,5]. Much of what we know about host factors that promote efficacious immune responses has been gleaned from studies of influenza virus infection or vaccination. This is due to both the availability of influenza virus infected/vaccinated individuals for study annually, as well as the fact that the diversity of the influenza virus vaccine types (e.g., inactivated, live-attenuated, adjuvanted, etc.) provide a useful opportunity for comparing different vaccine formulations.

**Figure 1:**
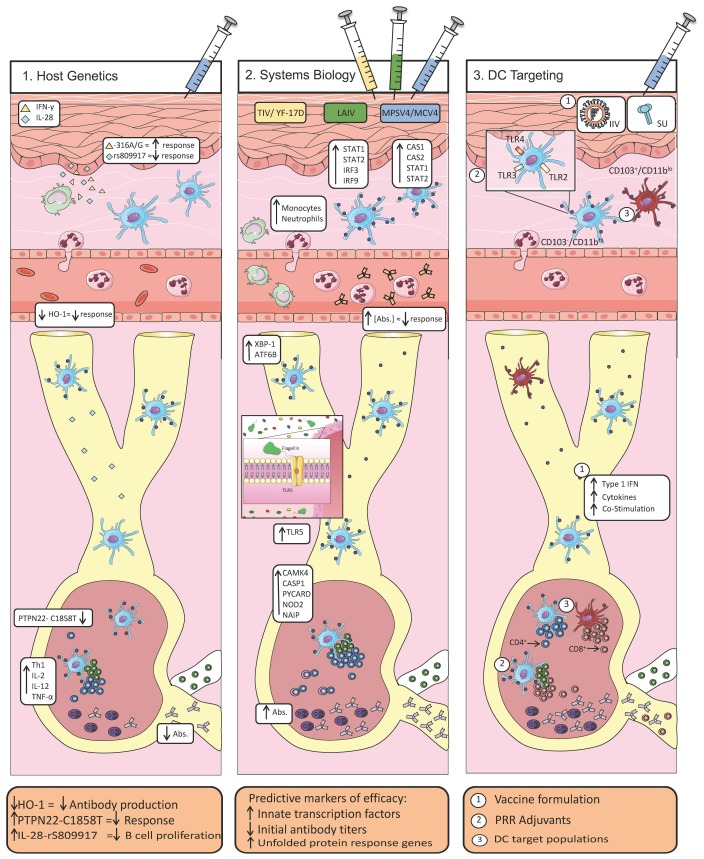
The impact of host factors in responses to vaccination. (Left) Polymorphisms in host genes have been shown to play a role in the response to vaccination. This has been demonstrated most extensively for genes associated with the production of antibodies. However, polymorphisms can also result in the augmentation or reduction of a protective response through other pathways. For example, reduced expression of heme oxygenase-1 (HO-1) has been shown to correlate with reduced antibody titers in response to vaccination. Likewise, polymorphisms that result in increased expression of IL-28 (and consequently, Th1-associated cytokines), or the presence of the C1858T mutation in the PTPN22 gene have been associated with abrogated antibody production and impaired immune responses. (Middle) Systems biology studies have the potential to identify signatures of protective responses. Effective vaccine responses induced by YF-17D, LAIV, TIV, polysaccharide-containing vaccines or conjugate vaccines increase the number of gene transcripts associated with the innate immune system including: *STAT1*, *STAT2*, *IRF3*, *IRF9*, *TNFRSF17*, *CAS1*, and *CAS2*. Interestingly, both the microbiome (by way of flagellin-induced TLR5-signaling) and the unfolded protein response (*XBP-1* and *ATF6B*) have also been shown to play a role in the protective response induced by TIV. At the same time, multiple studies reported that high pre-vaccination antibody titers correlated with a diminished response to vaccination. (Right) Coordinating innate and adaptive immunity is a critical determinant of efficacious responses. Targeting of distinct populations of DCs has been shown to differentially affect the downstream production of antibodies, and vaccine formulations can be modified to preferentially target these populations. For example, CD103^+^CD11b^lo^ DCs preferentially prime CD8^+^ T cells while CD103^-^CD11b^hi^ DCs more effectively prime CD4^+^ T cells. Finally, the addition of TLR agonists to adjuvants/vaccine preparations can act to increase antigen presentation and antibody production by enhancing innate immune signaling pathways.

While innovative antigen engineering strategies have pushed us closer than ever before to realizing “universal” influenza virus vaccines by targeting conserved epitopes, these strategies alone will not solve all of the problems that currently limit vaccine efficacy, including the problem of suboptimal immunogenicity [6]. Only by understanding and exploiting the host factors and pathways that are required for efficacious responses will novel vaccine strategies succeed in protecting the global population from future influenza virus epidemics and pandemics. In this paper, we review recent data that have shed light on host factors and pathways that are important for generating efficacious immune responses against the influenza virus (see Figure 1). We focus on studies of host polymorphisms, systems biology investigations of influenza virus vaccine responses, and the integration of innate and adaptive branches of immunity by dendritic cells (DCs). We discuss how these host pathways might be exploited for enhancing the efficacy of novel vaccines and we highlight gaps in understanding that should be a priority for future studies.

## 2. Host Determinants of Efficacious Vaccine Responses

### 2.1. Host Polymorphisms Influence the Development of Protective Immunity

Modern advances in technology and affordability of DNA sequencing have facilitated rapid leaps in the understanding of how host genetics influence the immune response. The identification of polymorphisms that result in augmented or impaired protective immune responses can provide significant insight into the pathways required for optimal immunity. In the context of influenza virus, potent neutralizing antibody responses are currently the best correlation for protection [7]. Therefore, polymorphisms that directly influence antibody quality and/or quantity are expected to have a profound influence on developing protective immunity against influenza virus. An interesting and somewhat unexpected example that has been described in both humans and mice is heme oxygenase-1 (HO-1), which was demonstrated to result in impaired antibody production when expression was reduced [8]. A recent article by Linnik and Egli [9] reviewed many more polymorphisms that impact antibody responses, such as SNPs in *IL6*, *IL12B*, and *IL1R1* [10]. Therefore, we will not discuss those in detail here. However, there is extensive evidence demonstrating that coordination between the innate and adaptive branches of immunity are also essential for developing efficacious responses. 

Polymorphisms in genes encoding interferons (IFN) and interleukins (IL) have received substantial attention for their effect on the host response to vaccination. IFN-γ has a particularly strong correlation with efficacy of vaccination for not only influenza virus but also for many other vaccines. A recent study has demonstrated that IFN-γ levels are elevated as soon as seven hours post trivalent inactivated influenza virus vaccine (TIV) administration and peak at approximately 24 h. However, the sample size of this study was somewhat limited (*n* = 20) [11]. Furthermore, IFN-γ has been reported to function as an important regulator in the balance between Th1 and Th2 responses and may prove to be a potentially attractive target for vaccine development. Additionally, it may be associated with the modulation of the immune response [2,12]. One particular member of the IFN-γ family, IL-28B, has been specifically implicated in the Th1/2 balancing role of IFN-γ. The rs809917 polymorphism in IL-28B (TG or GG minor-allele as compared to the major-allele consisting of TT) results in decreased expression of the protein and has been associated with increased rates of seroconversion following influenza vaccination. Furthermore, IL-28B restricted B cell proliferation and antibody production in vitro. It also resulted in an elevation of Th1-associated cytokines with a corresponding decrease in Th2 cytokines [12]. More evidence of the importance of a balanced immune response for maximal vaccine immunogenicity comes from a study of the common autoimmune mutation, C1858T, in the *PTPN22* gene. *PTPN22* encodes for lymphoid tyrosine phosphatase (LYP). Patients carrying this mutation displayed impaired immune responses following TIV influenza vaccination, which are characterized by decreased CD4^+^ T-cell expansion and antibody avidity [13].

Antigen transport and processing are critical pathways required for mounting adaptive immune responses. An integrative genomic analysis involving genetic, transcriptional, and immunological data reported that individual variation in the expression levels of proteins involved in these pathways may impact vaccine efficacy [14]. Similarly, antigen presentation is also an important determinant of vaccine immunogenicity [15]. Variation in HLA alleles have been reported to correlate with antibody titers after influenza virus vaccination. HLA-DRB1*04∶01 and HLA-DPB1*04∶01 have been correlated with seroprotective responses while HLA-DRB1*07 was enriched in non-responders [16,17]. Although analyses of this nature are extremely challenging to perform in humans, the development of the collaborative cross mouse model has facilitated the study of genetic variation in a tightly-controlled experimental system [18]. These studies have exploited the power of quantitative trait loci (QTL) mapping to identify genes associated with “high” responders and “low” responders following viral infection. Many genes that are known to play a role in viral infection, such as interferon-stimulated genes (ISGs, e.g., Mx1) have been reported in these studies in addition to novel genes that may not have been predicted to play a role in the response to infection, such as *MTA1* and *THNSL2*. These genes have roles in metastasis and the synthesis of threonine, respectively [19,20,21,22,23,24]. It is these unexpected genes, in particular, that could provide important new information into the pathways required for optimal immune responses.

### 2.2. A Systems-Level View of Vaccine Efficacy

Alongside sequencing technology, which has facilitated the identification of polymorphisms that affect the immune response to influenza virus, systems biology studies are beginning to provide a clearer understanding of the multi-dimensional immunological mechanisms that are induced in response to vaccination, and the signatures that are predictive of protective post-vaccination responses [25]. Understanding the parameters that define vaccine efficacy can be exploited to improve the immunogenicity of existing vaccines and inform the design of next-generation vaccines.

A variety of vaccine formulations against influenza virus have been approved by the U.S. Food and Drug Administration, of which the most commonly administered are the inactivated influenza vaccines (trivalent and quadrivalent) and the live-attenuated influenza vaccines (LAIV) [26]. New formulations including adjuvanted and recombinant hemagglutinin (HA) vaccines have entered the market more recently [27]. While each vaccine formulation is capable of conferring protection, the nature of the immune response elicited by each formulation differs and global features of efficacy are not well established. Importantly, influenza vaccines also differ in their route of administration. While inactivated vaccines are administered via intramuscular injection, LAIV is delivered directly at mucosal surfaces as a nasal spray. Using systems biology approaches, it is possible to define multi-parametric signatures of vaccine efficacy that are either global or formulation-specific.

The Yellow Fever Virus (YFV) YF-17D vaccine is among the most effective vaccines ever developed for use in humans. A single dose is capable of conferring lifelong protection from YFV in over 99% of individuals [28]. Using YF-17D as a prototype for defining an efficacious immune response to vaccination, the Pulendran laboratory published a seminal study in 2009 in which they demonstrated that systems biology could be used to define immunological signatures that correlate with vaccine immunogenicity [29].

Other groups have since used this approach to characterize the immune response to Influenza vaccination and infection. In 2011, Nakaya et al. published a comparison of individuals’ immune signatures after vaccination with LAIV or TIV [30]. These vaccines differ in both their formulation (live-attenuated vs. inactivated) and their route of administration (intranasal vs. intramuscular). In contrast to TIV, which is known to elicit robust seroconversion in peripheral blood, LAIV typically results in weaker seroconversion but is thought to elicit more robust local/mucosal immunity [31]. Microarray analysis of peripheral blood mononuclear cells (PBMCs) following LAIV vaccination revealed up-regulation of type 1 interferon and cytokines generally associated with the innate immune system, in addition to *STAT1*, *STAT2*, *TLR7*, and *IRF3* [30]. Interestingly, these findings were similar to those found using transcriptome analysis following vaccination with YF-17D, which is also a live-attenuated vaccine [29]. TIV, which resulted in more robust seroconversion, was enriched for gene signatures characteristic of DCs and B cells and other genes associated with the unfolded protein response (e.g., *XBP-1* and *ATF6B*), which are known to augment antibody production [30]. *CAMK4* expression was also reported to have predictive value in determining vaccine immunogenicity. CaMKIV kinase plays a role in a variety of immunological processes and high *CAMK4* expression on day 3 post vaccination was negatively correlated with a strong antibody response [30]. Vaccination of Camk4^−/−^ mice further supported this observation since these mice showed higher antibody responses to TIV vaccination when compared with wildtype mice. Genes that previously had not been linked to the antibody response, such as *TLR5*, *CASP1*, *PYCARD*, *NOD2*, and *NAIP* were also identified [30]. While PBMCs are a convenient source for analysis of vaccine-induced responses, caution must be taken in how studies of this nature are interpreted. PBMCs may indeed provide useful “signatures” of vaccine immunogenicity and/or efficacy, but the specific responses measured from these cells cannot be extrapolated to the immune system as a whole. As such, the genes and pathways required for optimal vaccine responses in other tissues (e.g., lymph nodes and the musoca) may be very different from those measured in the periphery. 

The unexpected role of TLR5—a pattern recognition receptor (PRR) that binds bacterial flagellin—in promoting influenza virus vaccine immunogenicity stimulated a follow-up study aimed at defining the role of TLR5 in mounting a protective humoral response to TIV vaccination [32]. Consistent with the earlier study in humans, TLR5^−/−^ mice had a significant reduction in TIV-specific IgG and IgM produced following vaccination [32]. However, this was not due to TLR5 signaling in response to the vaccine itself. Instead, the microbiome was implicated in the TLR5-mediated antibody response to TIV. Mice whose microbiome was depleted by treatment with antibiotics prior to vaccination exhibited diminished antibody responses to the vaccine [32]. While TLR5 was also found to be important for the IPOL polio vaccine, depleted commensal microbiota did not affect responses to YF-17D or Tdap (tetanus-diptheria-pertussis) vaccines [32]. Taken together, these studies suggest that TLR5-mediated sensing of microbiome-derived flagellin affected vaccine immunogenicity in a context-dependent manner. By extension, changes in the composition of the microbiome that occur as a result of aging or illness may affect the quality of response to certain vaccines.

Several of the B cell transcripts reported by Nakaya et al. following TIV vaccination (ex. *XBP-1* and *TNFSFR17*) were also detected in a later study along with *CXCR3* [33]. Consistent with the hypothesis that certain signatures of vaccine immunogenicity may transcend diverse vaccine types, a comparative study of protective gene signatures was conducted following vaccination with polysaccharide-containing vaccines. Conjugate vaccines reported upregulation of genes conserved between those vaccines and genes previously-associated with protective responses towards TIV, LAIV, and YF-17D. These included *TNFRSF17* and factors associated with the innate immune response such as *OAS1*, *OAS2*, *STAT1*, and *STAT2* [29,30,34].

The importance of innate immunity in promoting robust vaccine responses was reinforced by Obermoser et al. who reported an increase in IFN transcripts (*STAT1*, *IRF9*) one day following influenza vaccination [35]. At this time, innate immune cells including neutrophils and monocytes were abundant populations in the blood [35]. The innate immune response has also been implicated in the incongruent antibody responses observed in men and women following vaccination with TIV [36]. Higher levels of testosterone have been reported to antagonize NF-κB, which suppresses FOS/JUN and alters the production of pro-inflammatory cytokines involved in priming a protective immune response [36]. Variation between the sexes in response to vaccination has not only been observed in the context of influenza virus, but also in YFV, mumps, measles, rubella (MMR), and hepatitis [36]. In general, women have been reported to mount more robust immune responses and were shown to have increased serum antibody titers following vaccination compared to men. Many of these differences in responses between men and women have been associated with hormone levels. However, the mechanisms governing sex-specific and gender-specific differences remain poorly understood and warrant further investigation [37].

Older individuals are among those at the highest risk during flu season and have been shown to respond poorly to most current influenza virus vaccine formulations [38]. Dampening of the innate immune response following vaccination was shown to be a potential cause of this poor response in individuals over the age of 65 [38]. Significant decreases in the expression in early response genes *TBX21*, *CD38*, *CD28*, *HDAC4*, *IFN-γ*, and *CXCL10* were observed in older adults [38]. Adding adjuvants to vaccines is a well-documented strategy for improving immunogenicity, which may be especially useful in populations like the elderly who often have dampened innate responses. Adjuvanted TIV (ATIV), containing MF59 (a squalene-based adjuvant) produced a stronger antibody response and elevated expression of innate immunity-associated genes [39]. ATIV also enhanced the recruitment and activation of antigen presenting cells (APCs) and CD4^+^ vaccine-specific lymphocytes [39]. However, an even more robust antibody response was observed following vaccination with ASO3, which is another squalene-based adjuvant [40]. An increase in PBMC transcripts related to innate immune activation were observed again in genes associated with the interferon response and genes associated with APCs [40]. 

Tsang et al. compared responses elicited by the 2009 seasonal and pandemic (pH1N1) vaccines. Consistent with earlier studies, interferon-related genes were upregulated on the first day post-vaccination and the frequency of innate immune cells such as monocytes and plasmacytoid dendritic cells (pDC) were also elevated [41]. By the seventh day post-vaccination, the response signature transitioned to genes associated with the adaptive response, including plasmablast formation, endoplasmic reticulum stress, and N-glycan biosynthesis [41]. A number of cell populations, including CD4^+^ and CD8^+^ T cells, as well as naïve and memory B cells, showed an increase in frequency between the first and seventh day. Interestingly, the authors were able to identify post-vaccination cell populations that were strongly correlated with robust seroconversion, including myeloid DCs, activated T cells, and strong interferon signaling [41].

Strong induction of vaccine-specific antibody titers following vaccination is currently regarded as a measure of vaccine effectiveness. However, high titers of vaccine-specific antibodies pre-vaccination can be detrimental to the vaccine response [42]. It has been proposed that pre-existing antibodies may cause epitope-masking, which prevents the recognition and expansion of B cells specific to those epitopes [42]. The effect of pre-existing antibodies is particularly important in the context of seasonal vaccination since pre-existing antibodies may sometimes confer protection to previously-encountered strains at the expense of mounting effective responses against new strains present in the vaccine [43,44]. Interference caused by pre-existing antibodies also plays an important role in the timing and frequency of vaccination. When vaccines are administered in close proximity, elevated antibody titers generated in response to the first vaccination may interfere with the response to the second vaccination.

Despite differences in vaccine formulations and study methodologies, these reports highlight the critical importance of innate immune activation as an early post-vaccination event required for optimal vaccine immunogenicity. Strategies to maximize early innate immune responses while minimizing adverse reactogenicity should be a focus for developing novel vaccines and adjuvants.

### 2.3. Coordination of Innate and Adaptive Immunity via Dendritic Cells

Clearly, coordination of the innate and adaptive branches of the immune system is essential for mediating optimal immune responses against vaccines and infections themselves. This coordination is mediated by specific subsets of cells present in the host. DCs serve as an essential link between innate and adaptive immune responses by acting as APCs and by coordinating the two branches of the immune system [45,46,47]. DCs sense pathogens or vaccine antigen, collect information about the nature of the foreign substance, and subsequently, integrate that information to shape adaptive immune responses [48]. For initiating CD4^+^ T cell and CD8^+^ T cell responses following influenza vaccination adjuvanted with TLR ligands, direct sensing of pathogen-associated molecular patterns (PAMPs) by DCs is a crucial step [49]. These functions of DCs make them excellent targets for rational vaccine design against influenza virus and other pathogens [48,50]. However, there are many different subtypes of DCs and each subset has specific functional properties that must be considered when choosing a target population. For example, in mice, CD103^+^CD11b^lo^ DCs efficiently prime CD8^+^ T cells at the early stage of influenza virus infection while CD103^−^CD11b^hi^ DCs prime CD4^+^ T cells at steady-state conditions. Therefore, strategic targeting of specific DC populations in order to facilitate robust and balanced immunity is a promising approach for novel vaccine formulations. However, more detailed studies are needed in humans to better characterize analogous populations.

The IL-1 pathway plays a pivotal role in activating DCs after either influenza infection or vaccination [49]. In fact, secretion of IL-1α, as an alarmin, and IFN-β and MCP-1 by lymph node macrophages activate DCs. The IL-1 pathway in the lymph nodes enhances DC antigen presentation and improves antibody-mediated responses against influenza virus vaccines [51]. Another example of the importance of innate immune responses in the shaping of adaptive immune responses via DCs was highlighted by Lupfer et al. in 2014. This group reported that Nod2^−^/^−^ DCs are incapable of priming CD8^+^ T cells and were susceptible to influenza virus infections [52]. Lung tissue-resident memory CD8^+^ T cells, which is a self-sustaining population, efficiently interfere with influenza virus replication. This cell population can kill virus-infected cells, recruit other cells to the site of infection, and therefore, limit viral replication. Antigen presentation by local DCs and activation of the transforming growth factor-β (TGF-β) pathway is involved in the differentiation of tissue-resident memory CD8^+^ T cells [53,54]. Emerging evidence suggests that immunization with antigen coupled to monoclonal antibodies (mAbs) specific to receptors present on DCs could be an efficient way to initiate the differentiation of lung tissue-resident memory CD8^+^ T cells. This represents a valid strategy for development of new vaccines against influenza virus [55].

Importantly, the responses of DCs to different vaccine formulations(e.g., whole inactivated virus (WIV) and subunit (SU) influenza vaccine preparations) are distinct. For example, the WIV influenza vaccine has been shown to activate a different gene expression signature in DCs than a SU vaccine. The WIV influenza vaccine induced higher expression of genes related to antigen processing, antiviral responses, and regulation of leukocyte responses in DCs compared to the SU vaccine [56]. Adjuvants have been employed to increase respiratory DC functions and CD8^+^ T cell priming. It has been demonstrated that the mucosal administration of polyI:C, a TLR3 ligand, after vaccination with cold-adapted influenza vaccine significantly increased the activation and migration of antigen-bearing TLR3^+^ CD103^+^ respiratory DCs to the mediastinal lymph nodes, the generation of influenza-specific CD8 T cells, and the production of neutralizing antibodies [57].

Together, these studies highlight the essential role of DCs in coordinating the innate and adaptive branches of the immune system. Our current knowledge for the role of different subsets of DCs in viral infections provides a platform for designing DC-tailored vaccines that may serve to enhance vaccine efficacy and durability of response.

## 3. Discussion

The need to develop more efficacious vaccines against influenza virus is pressing. On average, the efficacy of seasonal trivalent inactivated influenza vaccines is around 60%. However, this has been much lower in several recent H3N2-dominated seasons [58,59]. This suboptimal protection compromises both the health of the population and undermines public confidence in the utility of influenza virus vaccines, which leads to lower overall coverage [60]. While new manufacturing practices (e.g., cell-based vaccines) and “universal” influenza virus vaccine platforms will reduce problems associated with egg-adaptations and the breadth of protection, they alone will not overcome the issue of suboptimal responses to vaccine antigens. Therefore, understanding host factors and pathways that are required for, or predictive of, efficacious responses to vaccines is essential.

It is clear that coordinating the innate and adaptive branches of the immune system is critical for achieving robust vaccine responses. Natural infection and, by extension, live-attenuated vaccines typically elicit strong innate immune responses due to the generation of PAMPs and “danger signals” associated with viral replication. However, subunit vaccines, which are usually compromised of purified recombinant protein that is intrinsically less immunogenic, often require adjuvants to strengthen immunogenicity. While the mechanisms-of-action of adjuvants vary substantially (and in some cases, are incompletely understood), most work by stimulating more robust innate immune responses [61]. 

Several studies underlined the potential application of PRR agonists as adjuvants for influenza vaccines [62]. However, until very recently, fears related to adverse reactogenicity stimulated by adjuvants containing PRR agonists have hindered regulatory approval. Most current adjuvant systems contain either alum or squalene-based oil-in-water emulsions, which do not directly engage PRRs. However, AS04 (GlaxoSmithKline), which was licensed in 2005, is used in human papillomavirus and hepatitis B virus vaccines and is composed of alum-adsorbed TLR4 agonist (monophosphoryl lipid A) [63]. Over the course of the past decade, the vast potential for adjuvants to improve vaccine efficacy and reduce antigen dose has been more fully realized. This, combined with the depth of knowledge that has accumulated pertaining to the safe delivery of these compounds, is likely to catalyze an extensive and much-needed expansion of approved adjuvant technologies in the years to come.

## 4. Conclusions

Perhaps the greatest obstacle to definining the role of host factors in influenza virus vaccine responses is the intrinsic difficulty of comparing between discrete studies. These comparisons are confounded by changes in vaccine strains from year-to-year, differences in vaccine formulations (e.g., inactivated, live-attenuated, subunit), and differences in sampling and analytical methodologies. Therefore, it will be important to standardize the timing, nature (e.g., whole blood, PBMCS, and serum), and methods of analysis used in large-scale studies of vaccine responses. This will facilitate the identification of factors, which are vaccine formulation-specific versus those that may apply to multiple vaccine types. Nevertheless, important information can be gleaned from comparisons of the effective responses elicited in healthy individuals versus responses that are ineffective in specific high-risk populations. In addition to a more sophisticated use of adjuvants to boost specific qualities of the immune response, differences in the responses elicited based on vaccine formulation and/or route of administration might also be exploited to overcome particular deficiences. For example, older adults who suffer from a decline in T cell function as a result of exhaustion or anergy might benefit from live-attenuated vaccine formulations that more effectively boost/stimulate T cell responses. Similar strategies could be employed to compensate for blunted responses experienced by patients treated with immunosuppersive drugs.

Simply identifying host factors/pathways important for efficacious responses is not sufficient to improve future vaccine design. We must continue to pursue basic experimental studies to probe the mechanistic basis for how these factors/pathways shape the immune responses. Only through “gnothi seauton” (knowing thyself) can we hope to protect ourselves by improving influenza virus vaccine design in the future.
